# Moths

**Published:** 1890-08

**Authors:** 


### MOTHS.

We take the following timely article on one of the most destructive of our household pests, from *The Housekeeper.*

The moth-miller lays her eggs in the best feeding ground she can find for the future life of the worm, which feeds on wool, hemp, fur, and feathers. This worm then spins its tiny cocoon and retires, to emerge again as a moth-miller. The latter makes her appearance between the middle of April and the last of June, and if the eggs are in the garment when put away it will be damaged in the fall no matter how many preventives have been used. The best precaution is to hinder the moth-miller from depositing eggs where the worm can do damage.

Give the garments to be put away, and which can not be washed, a thorough shaking and brushing, and expose them to the sun's rays a few hours. Then put them into paper flour sacks ; fold the edges of the sack and paste over it a thickness of muslin or paper, thereby sealing it so securely that the moth-miller can not get in. Another way to outwit her is to paper a large box on the inside, fill it with woolen garments, fit on the cover, then paste paper all over the outside.

To protect furniture and carpets, saturate strips of red flannel with a solution of arsenic, and lay them under the edge of the carpets, inside the lining of the furniture covers. The worm will eat of them and die.

Moths are especially fond of baby's clothes. If the miller finds a spot where milk has been, she will deposit her eggs there. A wool mattress is another place for which she shows great partiality, and it is hard to prevent her from depositing eggs somewhere in it. The best remedy is to prop the mattress up on blocks of wood out in the yard, and set under it a dish of live coals on which you must sprinkle sulphur from time to time. Care must be used to prop it high enough not to become scorched.

Remember that whatever you use must be used in time to prevent the moth from depositing her eggs, as there are few things that will kill the worm. Oil of cedar, turpentine, camphor, oil of cloves or wintergreen will keep the worm away, but evaporate quickly, and should be used early and renewed often. They kill neither moth nor worm, and must be strong to be effective.

